# Glans dehiscence after severe hypospadias repair. Is it a real complication? Clues from a study in post-pubertal patients

**DOI:** 10.1007/s00383-023-05387-0

**Published:** 2023-02-03

**Authors:** Ludovica Durante, Filippo Ghidini, Francesco Panchieri, Eleonora Bovolenta, Vincenzo Bagnara, Ciro Esposito, Marco Castagnetti

**Affiliations:** 1 Paediatric Urology Unit, Department of Surgery, Oncology, and Gastroenterology, University of Padova, Padua, Italy; 2 Pediatric Surgery Unit, Policlinico “G.B. Morgagni”, Catania, Italy; 3 Division of Pediatric Surgery, Federico II University of Naples, Naples, Italy; 4 Department of Surgery, Oncology, and Gastroenterology, University of Padova, Via VIII Febbraio, 2, Padua, Italy; 5 Pediatric Urology Unit, Bambino Gesù Children Hospital and Research Center, Rome, Italy

**Keywords:** Hypospadias, Tabularized incised plate repair, Glans dehiscence, Quality of life, Puberty, Long-term outcome

## Abstract

**Introduction:**

Glans dehiscence (GD) is reportedly a common complication after proximal hypospadias repairs. However, the need for surgical correction is controversial. The aim of this study was to assess awareness, risk factors, and outcome of GD in post-pubertal patients. The agreement with surgeon assessment was also evaluated.

**Methods:**

The design was retrospective. All consecutive patients treated for proximal hypospadias between 2000 and 2011 were included. The presence of GD was self-reported, and the participants could optionally upload a photograph for surgeon assessment. Cosmetic and functional outcomes were assessed by validated questionnaires (HOSE, PPPS, KINDL^®^, IIEF-5). Results were compared between patients with and without GD.

**Results:**

Of 219 patients, 34 (16%) participated. Fourteen of them (41%) self-reported GD. Eighteen patients (8%) also uploaded a photograph and, in ten of them (56%), the surgeon noted the presence of GD with poor agreement [k = − 0.444 (95 CI − 0.856 to − 0.033)] with patient report. Patients self-reporting GD had had more frequently a penile curvature at diagnosis (12/14 = 86%, *p* = 0.01), and had undergone a single-staged repair (100% vs. 65%, *p* = 0.03). No difference was found in cosmetic and functional outcomes. Results were similar also comparing groups with and without GD as assessed by the surgeon.

**Conclusion:**

GD was a common finding after severe hypospadias repair. It was more common in case of surgeon assessment with poor agreement between patients and surgeons. GD did not prove to have clear clinical implications. Therefore, in our opinion, surgical repair of GD should be recommended only on patients request.

## Introduction

In a recent series, Snodgrass and Bush maintained the creation of glans wings fusion from meatus to corona of minimum 2 mm or greater, as an important goal of hypospadias repair [1]. As opposite, glans dehiscence (GD) is a separation of the glans wings approximated during hypospadias repair resulting in a meatal retrusion to the corona. Reportedly, GD occurs in 9–17% of patients after severe hypospadias repairs, and 4% of the those operated on for distal hypospadias [2]. Snodgrass et al. identified two independent risk factors for GD including proximal meatal location and a history of previous surgeries. In their experience, the former increased the risk for GD by 3.6-folds whereas the latter by 4.7-folds [2].

GD is a common indication for re-intervention in hypospadias surgery [3, 4]. The surgical repair of GD has been advocated for cosmetic reasons and to avoid a spraying pattern or a downward deflection of the urinary stream [2]. Nevertheless, the cosmetic relevance of GD for the patient has not been proven and GD, by reducing the resistance of the newly reconstructed urethra, might even improve the voiding pattern [3]. For this reason, also conservative management has been advocated as an option in these patients [3]. Consistently, our subjective impression is that often patients and their parents are unaware of the presence of GD.

This study aimed to assess patient awareness, risk factors, and outcomes of GD in post-puberal patients after primary repair of proximal hypospadias during childhood. To assess awareness, the agreement between surgeon and patient opinions about glans appearance was investigated. Risk factors, and long-term cosmetic and functional outcomes of GD were instead assessed comparing patients with GD and those with normal glans appearance. Long-term outcomes were assessed by validated patient-reported outcome (PRO) questionnaires, that at present seem to be the most useful tool in these patients [4–6].

Our hypotheses were that many patients experiencing GD might be unaware of this complication and that GD has little impact on long-term urinary function and patient perception of penis appearance.

## Materials and methods

### Study design

This was a retrospective and observational study. The study was approved by the institutional review board (IRB).

### Study population

The institutional database was searched in May 2021.

Inclusion criteria were (1) history of proximal or mid shaft hypospadias; (2) date of surgery between January 2000 and December 2011; (3) patient undergoing a primary repair; (4) repair performed by long tabularized incised plate urethroplasty (TIPU), onlay island flap, or two-stage free graft repair; (5) postoperative follow-up of 10 years or longer.

Patients with known physical or psychological impairment were excluded as potentially unsuitable to reliably answer the questionnaires.

Patient baseline characteristics, such as meatal position, presence of associated curvature, urethroplasty technique, and maneuvres for penile straightening, were assessed by review of the clinical notes.

All patients were treated by either of two board certified pediatric urologists (Fellows of the European Association for Pediatric Urology), with more than 5-year experience in the field.

### Study protocol

The study was carried out by an independent observer, with experience in pediatric urology, but not previously involved in the care of the patients. The patients and their legal guardians were contacted by telephone to obtain their consent to participate. The details of the study, together with the instructions and a link for the online questionnaire on Google Form^®^, were sent by email to those who agreed to participate. Participants were allowed 5 weeks to fill the online questionnaires. A remainder was sent to those who failed to return the questionnaires and further 5 weeks were allowed.

Instructions were given that the survey was completed only by the patient and never by the parents.

Participants were asked to self-assess the presence of GD. Participants were also invited to optionally upload a photograph taken on the ventral radius of the penis after pulling the phallus upwards toward the umbilicus. These pictures were independently assessed by two pediatric urologists to establish the presence of the GD.

Participants were then asked to self-administer the following validated questionnaires, (1) the hypospadias objective scoring evaluation (HOSE) [7], (2) the pediatric penile perception score (PPPS) [8], (3) the KINDL^®^ score [9], (4) the IIEF5 [10]. The first two questionnaires aimed to assess penile appearance and patient satisfaction about penile appearance, respectively. The KINDL^®^ score aimed to assess the health-related quality of life (QoL) according to the age of the participant. The IIEF5 was reserved only to participants older than 18 years for the evaluation of erectile function.

A set of additional non-validated questions was finally administered to collect further data, such as the number of surgical interventions, including the rate of complications, the presence of urinary symptoms such as a need for straining or post-voiding dribbling, the favourite voiding position (either sitting or standing), the desire to receive additional surgery to improve penile appearance, and the memory of surgical procedures performed during childhood.

### Groups, main outcome, and variables

To gauge generalizability of results to the study population, baseline characteristics between responders and non-responders were compared.

To gauge patient awareness of GD, agreement between patient self-assessment and surgeon assessment of provided pictures was determined.

To assess potential risk factors for GD and the influence of GD for the patient, results of the questionnaires and free text questions were compared between patients reporting GD and those who did not.

### Statistical analysis

The answers of the survey were gathered into a database created by Microsoft^®^ Excel. The categorical variables were reported as number (%) and the continuous variables were reported as their median value and inter-quartile range (IQR). For inter-observer agreement, Cohen’s kappa coefficient was calculated. For the comparison between the two groups, Fisher’s exact tests were used for categorical variables and Mann–Whitney *U* tests were used for the continuous ones. The statistical results were provided by IBM^®^ SPSS Inc. Version 26.0. *p* value ≤ 0.05 was considered statistically significant.

## Results

**Fig. 1:**
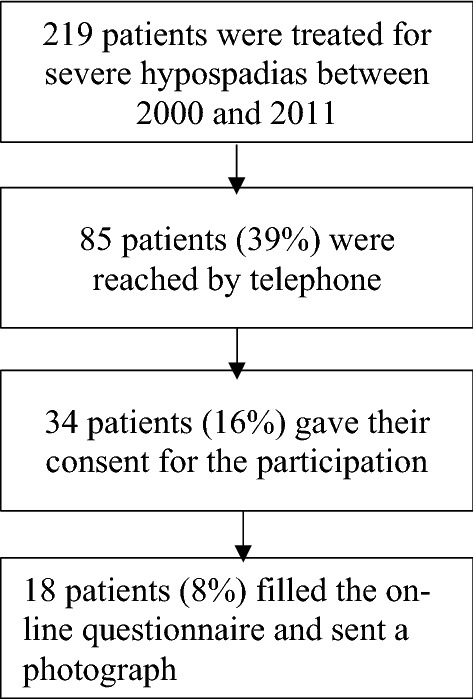
Response rate of the study population

Figure 1 displays the flow chart for patient selection.

Two hundred and nineteen patients matched the inclusion criteria for study. Eighty-five patients could be reached out. Their median age at surgery was 18 (IQR 14–25) months. Thirty-four of them answered the online questionnaire, yielding a response rate of 16%. Eighteen of the thirty-four patients (53%) also uploaded a photograph. No differences were found between participants and non-responders (Table 1). Of note, even though not significant, the complication rate was higher in participants (38% vs. 20%, *p* = 0.06). No differences were found in the type of complications.

**Table 1:** Characteristics of participants and non-responders

	Participants (*n* = 34)	Non-responders (*n* = 51)	*p* value
Age at intervention (median, IQR)	17 (14–4) months	18 (14–24) months	0.87
Length of follow-up (median, IQR)	15 (14–18) years	16 (14–17) years	0.96
Age at survey time (median, IQR)	17 (15–19) years	17 (15–20) years	0.89
Hypospadias according to Duckett’s Classification (*n*, %)	Mid-shaft 12 (35)	Mid-shaft 13 (25)	0.75
	Penoscrotal 10 (29)	Penoscrotal 20 (39)	
	Scrotal 10 (29)	Scrotal 15 (29)	
	Perineal 2 (5.9)	Perineal 3 (5.9)	
Associated curvature (*n*, %)	20 (59)	30 (59)	0.99
Associated scrotal anomalies (*n*, %)	7 (21)	7 (14)	0.40
Surgical technique (*n*, %)	Single-stage 27 (79)	Single-stage 44 (86)	0.40
	Two-stage 7 (21)	Two-stage 7 (14)	
Orthoplasty (*n*, %)	6 (17)	12 (24)	0.52
Surgical interventions per patient (median, IQR)	2 (1–2)	1 (1–2)	0.10
Complications requiring surgical corrections (*n*, %)	13 (38)	10 (20)	0.06
	Fistula 9 (69)	Fistula 6 (60)	0.33
	Stenosis 1 (7.7)	Stenosis 3 (30)	
	Others 3 (23)	Others 1 (10)	

Agreement about the presence of GD between patient self-assessment and surgeon’ opinion on the pictures uploaded was poor. Agreement was found in only 5 out of 18 cases (Cohen’s kappa coefficient − 0.444 (95 CI − 0.856 to − 0.033)).

Table 2 summarizes the risk factors in patients with vs. without GD. Patients self-reporting GD presented more frequently a penile curvature at diagnosis (12/14 = 86%, *p* = 0.01) and required a straightening procedure during hypospadias repair (5/14 = 36%, *p* = 0.02). Patients that did not refer GD had undergone significantly more commonly a staged repair (7/20 = 35%, *p* = 0.02). Complication rates were comparable between groups (*p* = 0.52).

**Table 2:** Baseline variables in patients with vs. without GD either self-reported by the patients or assessed by the surgeon

		GD	No GD	*p* value
GD self-reported by patients: 14 GD vs. 20 No GD	Age at intervention (median, IQR)	18 (16–24) months	17 (15–22) months	0.83
	Length of follow-up (median, IQR)	16 (14–19) years	14 (13–16) years	0.74
	Age at survey time (median, IQR)	17 (15–21) years	17 (15–18) years	0.43
	Hypospadias according to Duckett’s Classification (*n*, %)	Mid-shaft 5 (36)	Mid-shaft 7 (35)	0.85
		Penoscrotal 5 (36)	Penoscrotal 5 (25)	
		Scrotal 4 (29)	Scrotal 6 (30)	
		Perineal 0 (0)	Perineal 2 (10)	
	Associated curvature (*n*, %)	12 (86)	8 (40)	0.01*
	Associated scrotal anomalies (*n*, %)	2 (14)	5 (25)	0.45
	Surgical technique (*n*, %)	Single-stage 14 (100)	Single-stage 13 (65)	0.03*
		Two-stage 0 (0)	Two-stage 7 (35)	
	Orthoplasty (*n*, %)	5 (36)	1 (5)	0.02*
	Surgical interventions per patient (median, IQR)	1 (1–1.8)	2 (1.8–2)	0.02*
	Complications requiring surgical corrections (*n*, %)	4 (29)	8.0 (40)	0.49
		Fistula 2 (50)	Fistula 6 (75)	0.52
		Stenosis 1 (25)	Stenosis 0 (0)	
		Others 1 (25)	Others 2 (25)	
GD assessed by surgeon: 10 GD vs. 8 No GD	Age at intervention (median, IQR)	18 (14–27) months	16 (15–16) months	0.67
	Length of follow-up (median, IQR)	16 (13–18) years	14 (14–15) years	0.50
	Age at survey time (median, IQR)	17 (14–22) years	15 (15–17) years	0.42
	Hypospadias according to Duckett’s Classification (*n*, %)	Mid-shaft 1 (10)	Mid-shaft 3 (38)	0.69
		Penoscrotal 4 (40)	Penoscrotal 3 (38)	
		Scrotal 4 (40)	Scrotal 2 (25)	
		Perineal 1 (10)	Perineal 0 (0)	
	Associated curvature (*n*, %)	5.0 (50)	6.0 (75)	0.28
	Associated scrotal anomalies (*n*, %)	2.0 (20)	2.0 (25)	0.80
	Surgical technique (*n*, %)	Single-stage 7 (70)	Single-stage 8 (100)	0.19
		Two-stage 3(30)	Two-stage 0 (0)	
	Orthoplasty (*n*, %)	2 (20)	1 (13)	0.67
	Surgical interventions per patient (median, IQR)	2 (1–2)	1.5 (1–2)	0.76
	Complications requiring surgical corrections (*n*, %)	3 (30)	4 (50)	0.39
		Fistula 1(33)	Fistula 3 (75)	0.63
		Stenosis 1 (33)	Stenosis 0 (0)	
		Others 1 (33)	Others 1 (25)	

No difference was found in cosmetic outcomes, urinary symptoms, and quality of life between patients self-reporting GD vs. those who did not (Table 3). No difference was observed also considering the comparison of groups with and without GD as assessed by the surgeon (Table 3).

**Table 3:** Results of questionnaires in patients with vs. without GD either self-reported by the patient or assessed by the surgeon

		GD	No GD	*p* value
GD self-reported by patients: 14 GD vs. 20 No GD	HOSE score (median, IQR)	13 (12–14)	14 (13–15)	0.13
	PPPS score (median, IQR)	19 (15–23)	17 (17–18)	0.47
	Unsatisfied for cosmetic outcome (*n*, %)	5 (36)	11 (55)	0.27
		Size 3 (60)	Size 3 (27)	0.76
		Curvature 0 (0)	Curvature 3 (27)	
		Glans 2 (40)	Glans 2 (18)	
		Meatus 0 (0)	Meatus 1 (9.1)	
		Scars 0 (0)	Scars 2 (18)	
	Recall of the surgery (*n*, %)	7 (50)	10 (50)	1.00
		Glans 3 (43)	Glans 2 (20)	0.43
		Circumcision 3 (43)	Circumcision 4 (40)	
		Scars 1 (14)	Scars 4 (40)	
	Self-reported micturition symptoms (*n*, %)	1 (7.1)	2 (10)	0.77
	Self-reported anomalous micturition patterns (*n*, %)	6 (43)	12 (60)	0.32
		Spray flow 1 (17)	Spray flow 2 (17)	0.71
		Weak flow 2 (33)	Weak flow 2 (17)	
		Dribbling 3 (50)	Dribbling 8 (67)	
	KINDLE score (median, IQR)	65 (63–66)	65 (60–68)	0.53
		Available for 9 patients	Available for 14 patients	
	EuroQoL 5 score (median, IQR)	18 (18–18)	18 (18–18)	0.71
		Available for 5 patients	Available for 6 patients	
	IIEF-5 score (median, IQR)	25 (23–25)	7.5 (1.3–21)	0.27
		Available for 5 patients	Available for 6 patients	
GD assessed by surgeon: 10 GD vs. 8 No GD	HOSE score (median, IQR)	13 (12–15)	13 (12–14)	0.97
	PPPS score (median, IQR)	17 (15–17)	17 (16–22)	0.35
	Unsatisfied for cosmetic outcome (*n*, %)	7 (70)	4 (50)	0.39
		Size 3 (43)	Size 3 (75)	0.92
		Curvature 2 (29)	Curvature 0 (0)	
		Glans 1 (14)	Glans 1 (25)	
		Meatus 1 (14)	Meatus 0 (0)	
		Scars 0 (0)	Scars 0 (0)	
	Recall of the surgery (*n*, %)	7 (70)	3 (38)	0.17
		Glans 2 (29)	Glans 1 (33)	0.96
		Circumcision 3 (43)	Circumcision 1 (33)	
		Scars 2 (29)	Scars 1.0 (33)	
	Self-reported micturition symptoms (*n*, %)	2 (20)	0 (0)	0.36
	Self-reported anomalous micturition patterns (*n*, %)	9 (90)	4 (50)	0.06
		Spray flow 2 (22)	Spray flow 1 (25)	0.45
		Weak flow 0 (0)	Weak flow 1 (25)	
		Dribbling 7 (78)	Dribbling 2 (50)	
	KINDLE score (median, IQR)	64 (60–68)	65 (63–66)	0.94
		Available for 6 patients	Available for 7 patients	
	EuroQoL 5 score (median, IQR)	18 (18–18)	–	–
		Available for 4 patients		
	IIEF-5 score (median, IQR)	25 (25–25)	–	–
		Available for 4 patients		

## Discussion

In present series, GD was reported by 41% of participants. When the presence of GD was assessed by the surgeon on penile pictures, it was noted in 50% of cases and agreement between surgeon and patient assessment was poor. Patients with self-reported GD had more often associated ventral curvature at the outset and had undergone more often a single-stage repair. Cosmetic and urinary outcomes were not significantly different in patients with and without GD. GD did not seem to influence sexual function and quality of life.

The prevalence of GD in present series was much higher than in the literature, where it is reported to occur in up to 17% of patients undergoing proximal hypospadias repair in childhood [2]. A selection bias might account for this as unsatisfied patients might be more keen to participate in the study to seek advice. Another possible explanation, however, is that GD is generally underreported because patients do not complain about it and, therefore, surgeons often overlook it. Consistently, the agreement between surgeon and patients on the presence of GD in present series was poor, which is consistent with previous studies [11].

In terms of risk factors for GD, previous studies have found this complication to be more common in severe vs. distal repairs [2], and this is a major reason why we included only severe cases in this study. The small glans size observed in in two thirds of patients with severe hypospadias has been suggested to account for the higher GD rate in severe hypospadias [12, 13]. Consistently, recent studies have investigated the role of preoperative hormonal stimulation with testosterone to promote glans growth and prevent GD [14]. Unfortunately, testosterone stimulation proved effective in determining a twofold increase in glans width [13, 14], but this did not result in a reduced rate of GD eventually [13]. Therefore, other factors should come into play. In present experience, patients reporting GD had more commonly an associated curvature at presentation and underwent a single-stage repair. Regarding the former, associated curvature is a known marker of severity of hypospadias, which determines the complexity of the repair, the risk of complications, and the final satisfaction [15–17]. Regarding the technique, present results corroborate our opinion that the staged approach is one of the most effective approaches to correct curvature and to prepare the glans for subsequent reconstruction [18–21].

In principle, glans reconfiguration is assumed to be important during hypospadias repair both for cosmetic and functional reasons. Regarding cosmesis, glans shape is a major item of the pediatric penile perception score (PPPS) [8]. As to urinary function, a normal glans reconfiguration is considered important for directing the urinary stream, as GD should result in urinary spraying or downward deflected of the urinary stream [2]. Present study does not support these assumptions as we find no significant difference in PPPS, HOSE score, urinary function, QoL, and sexual function between patients with and without GD, either self-reported or assessed by the surgeon. Consistently, a recent study investigating long-term outcomes after proximal hypospadias repair found that the position and the shape of the reconstructed meatus were not considered the main predictors for unsatisfactory outcomes, while the penile length and the ventral curvature were considered more significant [17]. At the same time, it is important to emphasize that theoretically meatal retrusion to the sulcus after GD might have some beneficial effects. The glans is the stiffest portion of the urethra; therefore, GD might improve urinary function, particularly when a long neo-urethra is fashioned, such as in proximal cases [3]. This might also result in a lower risk of urethral complications. This emphasizes, in our opinion, that the clinical relevance of GD should be gauged according to the severity of hypospadias. A successful glans reconstruction might be more relevant in distal repairs where surgery has expectedly a much lower complication rate and the cosmetic outcome is the main goal.

Despite the lack of difference in outcomes between patients with and without GD, in our opinion, the relevance of GD for post-pubertal patients should not be underestimated. Patients and families should be informed about the risk of GD and its possible surgical correction. Long-term follow-up should be ensured. The perception of the body image changes from childhood to puberty and poor cosmesis might acquire major relevance. Recently, Chang et al. found that patients unsatisfied for the appearance of the glans sought advice for surgical correction during adolescence [15]. This finding, together with the increased risk of recurrent ventral curvature in adolescents [15], supported the need for a long-term follow-up after hypospadias repair. Surgery is certainly warranted in patients suffering from an abnormal stream or those unsatisfied with penile appearance.

Present study has limitations. First, the sample size was limited and this might have impacted the detection of significant differences. The long timespan of follow-up might have affected sample size. Indeed, the older patients were difficult to reach. The low response rate has limited particularly the available information on quality of life and sexual function. Additionally, the retrospective design of the study might have further selected the participants, influencing the collection and the interpretation of the data.

To conclude, in our experience, GD was common in post-pubertal patients who underwent proximal hypospadias repair during childhood. For this reason, glans shape should be carefully assessed during long-term follow-up, although we find poor agreement between patient and surgeon assessment. Surgical correction should be offered, in our opinion, only to motivated patients with specific complains.

## Data Availability

Detailed anonymus data could be made available upon request to the corresponding author.
